# Supplementary material for: Olfactory coding in the antennal lobe of the bumble bee Bombus terrestris
Source: Sci Rep. 2021 May 26;11:10947. doi: 10.1038/s41598-021-90400-6 (PMC8154950; doi:10.1038/s41598-021-90400-6)
Supplement: Supplementary file 1 — Supplementary Information. [file 41598_2021_90400_MOESM1_ESM.pdf]

Supplementary information – Mertes et al.

Supplementary Figure legends

Figure S1. Odor quality coding depending on functional group or carbon chain length information – comparisons using pixelwise correlation coefficients between odor-response maps. **(A)** Similarity between presentations of the same or of different odorants. Activity maps are more similar (higher correlation coefficient) when the same odorant is presented, showing specific odor coding in l-ALT projection neurons ($p < 0.001$). **(B)** Odorants with the same functional group induce more similar activity patterns (higher correlation coefficient) than odorants with different functional groups ($p < 0.001$). **(C)** Odorants with the same chain length induce more similar activity patterns (higher correlation coefficient) than odorants with different chain lengths ($p < 0.001$). **(D)** Similarity between odorants depending on the difference in their number of carbon atoms. Pixelwise correlation coefficients decrease (i.e. response maps are more dissimilar) with increasing difference in the number of carbon atoms ($p < 0.001$; a *vs* b, $p < 0.01$; a *vs* c, $p < 0.001$; b *vs* c, $p < 0.001$).

Figure S2. Comparison of odor-response maps observed in two individual honeybee and bumble bee. The figure (left antennal lobe) presents four example odorants including all functional groups and carbon chain lengths tested in this study. To emphasize glomerular patterns, each map is scaled to its own maximum (red) and minimum (dark blue). The general arrangement of the patterns appears similar for some odorants (1-hexanol), less for others (octanal).

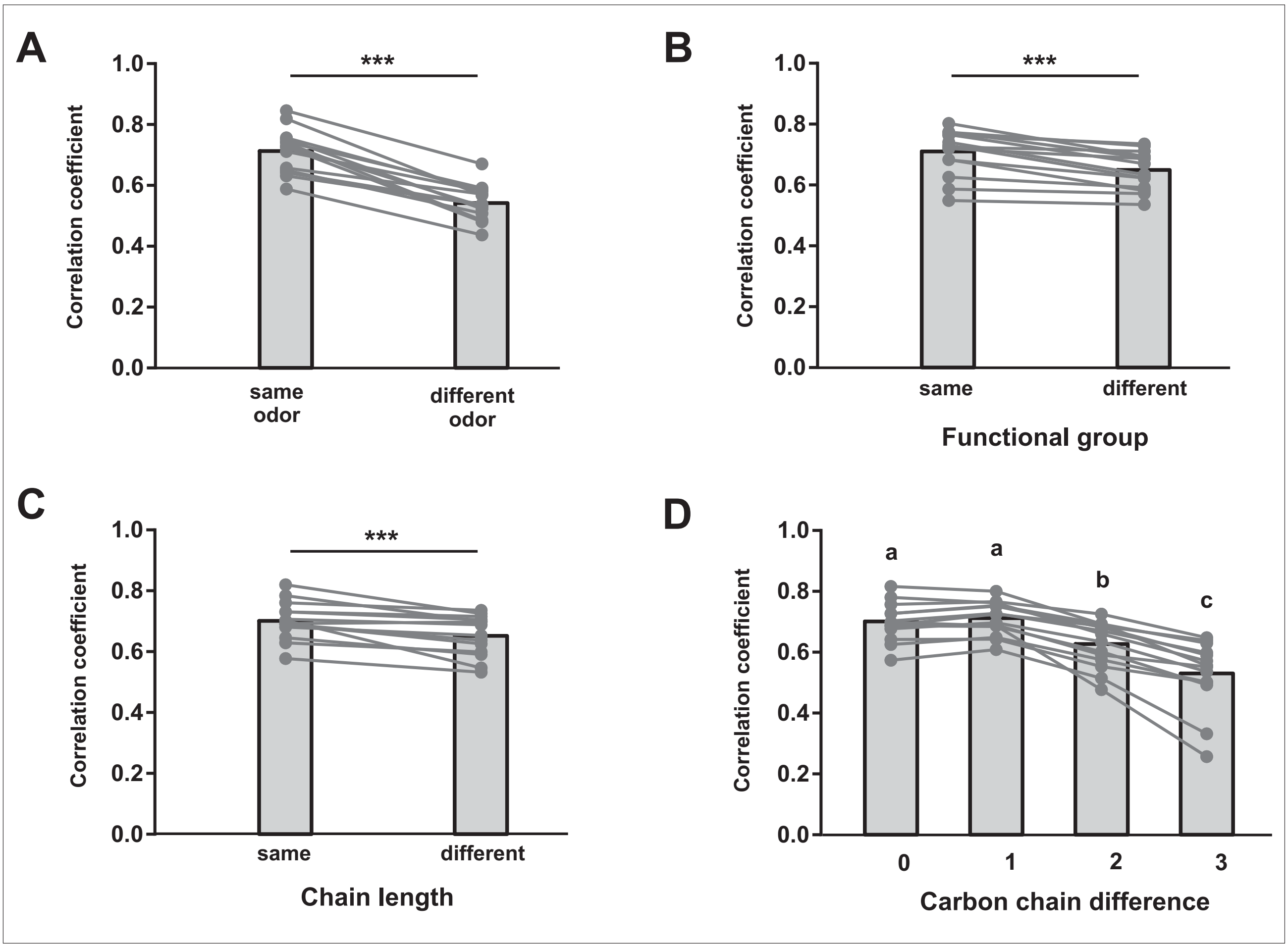
A
B
C
D
Correlation coefficient
1.0
0.8
0.6
0.4
0.2
0.0
***
same odor
different odor
same
different
Functional group
Chain length
a
a
b
c
0
1
2
3
Carbon chain difference

Figure S1

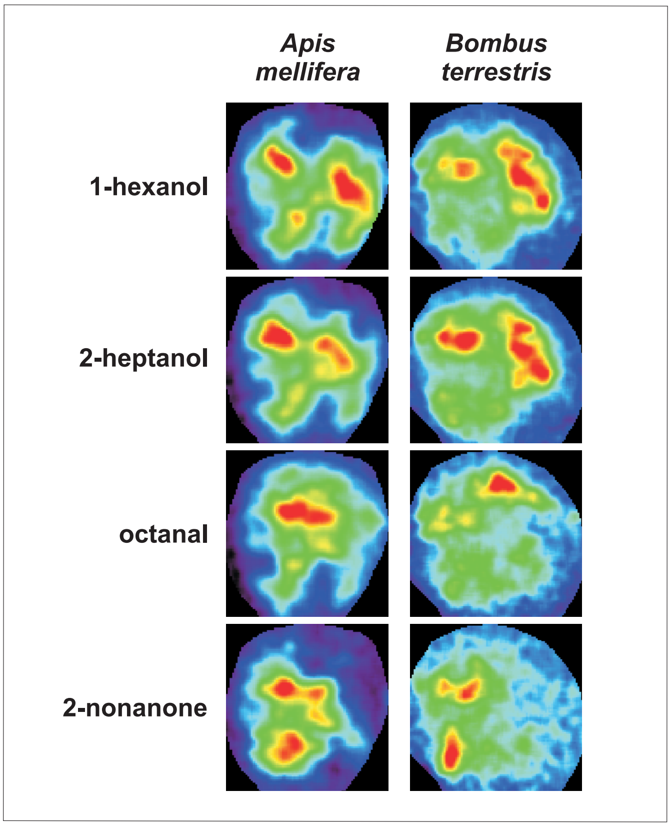
Apis mellifera
Bombus terrestris
1-hexanol
2-heptanol
octanal
2-nonanone

Figure S2
